# The complexities of salt taste reception: insights into the role of TMC4 in chloride taste detection

**DOI:** 10.3389/fnmol.2024.1468438

**Published:** 2024-09-25

**Authors:** Yoichi Kasahara, Masataka Narukawa, Yoshikazu Saito, Keiko Abe, Tomiko Asakura

**Affiliations:** ^1^Department of Applied Biological Chemistry, Graduate School of Agricultural and Life Sciences, The University of Tokyo, Tokyo, Japan; ^2^Department of Food and Nutrition, Kyoto Women's University, Kyoto, Japan; ^3^Toyo Institute of Food Technology, Hyogo, Japan; ^4^Department of Liberal Arts, The Open University of Japan, Chiba, Japan

**Keywords:** salty taste, chloride channel, TMC4, chloride taste, amiloride-insensitive

## Abstract

Although salt is an essential substance vital to life, excessive salt intake could cause various health issues. Therefore, new technologies and strategies should be developed to reduce salt intake without compromising taste. However, the underlying physiological mechanisms of salt taste reception is complex and not completely understood. Sodium chloride is a typical salty substance. It is widely believed that only sodium is important for the generation of salty taste. On the other hand, from a psychophysical perspective, the importance of chloride in salty taste has been indicated. Thus, understanding the mechanisms of both sodium- and chloride-tastes generation is necessary to completely comprehended the fundamentals of salt taste reception. However, the mechanism for detecting chloride taste has remained unclear for many years. Recently, we have identified transmembrane channel-like 4 (TMC4) as the first molecule that mediates the reception of chloride taste. TMC4 functions as a voltage-dependent chloride channel and plays an important role in the reception of the chloride taste by detecting chloride ions. In this mini-review, we first introduce the known reception mechanism of salty taste, and then discuss the roles of TMC4 in the salt taste reception. The finding of TMC4 may serve as a basis for developing new technologies and formulating strategies to reduce salt intake without compromising taste.

## Introduction

Taste is a sensory reception that is widely shared among many species. It receives stimulus from the external environment and transmits signals to the living organism through taste receptors. This suggests that taste is an initial trigger of physiological communication between the brain and internal organs. Taste is classified into five basic tastes (sweet, salty, sour, bitter, and umami) (Chandrashekar et al., 2006; Yarmolinsky et al., 2009). Taste receptors that detect sweet, sour, bitter, and umami tastes have been identified, and the details of their transmission are being clarified (Chandrashekar et al., 2006; Yarmolinsky et al., 2009). However, many uncertainties remain regarding the salt taste reception.

The typical substance representing salty taste is sodium chloride (NaCl). Excessive intake of sodium, found in NaCl, has been linked to various health risks, including hypertension and kidney dysfunction (Robinson et al., 2019). Therefore, the World Health Organization (WHO) recommends a daily sodium intake of 2.0 g/day (equivalent to 5.0 g/day of NaCl) (World Health Organization, 2012). However, despite understanding the need to control sodium intake, many people find it difficult to meet the WHO guidelines because they cannot suppress their craving for salty foods. In 2019, the mean global sodium intake of adults was estimated at 4.3 g/day (equivalent to 10.8 g/day of NaCl), which is more than double the WHO guidelines (World Health Organization, 2023). Therefore, new technologies and strategies should be developed to reduce sodium intake without compromising taste. However, the underlying physiological mechanism of salt taste reception is complex and not completely understood. If the molecular mechanisms underlying salt taste reception is elucidated, it could contribute to the development of salt taste enhancers that amplify salty taste even with small amounts of sodium, as well as the formulation of appropriate salt reduction policies.

## Mammalian peripheral taste reception

Taste reception occurs when taste substances are detected by the taste buds on the tongue. Taste buds, which consist of 50–150 taste cells, are mainly located in the fungiform, foliate, and circumvallate papillae of the tongue epithelium (Delay et al., 1986; Kinnamon et al., 1993; Yarmolinsky et al., 2009; Muchlinski et al., 2011). The fungiform papillae at the anterior part of the tongue are mainly innervated by the chorda tympani nerve, whereas the circumvallate and foliate papillae at the posterior part of the tongue are mainly innervated by the glossopharyngeal nerve, both of which transmit taste stimulus to the brain (Smith and St John, 1999; Ohla et al., 2019). Taste cells are classified into four types, I-IV, based on their morphological features and the molecular-markers they express (Kinnamon and Finger, 2019). Type II cells are involved in the detection of sweet, umami, and bitter tastes, whereas type III cells are involved in the detection of sour taste (Zhang et al., 2003; Huang et al., 2006; Matsumoto et al., 2011). Type I cells are thought to support the structure and metabolism of taste buds and assist in the function of taste cells, similar to the glial cells in neurons (Dvoryanchikov et al., 2009; Cherkashin et al., 2016; Guarascio et al., 2021; Rodriguez et al., 2021). Type I cells constitute approximately 50% of taste cells; however, their direct involvement in taste reception is not well understood (Ma et al., 2007; Yang et al., 2020). Type IV cells, also known as basal cells, are considered precursor cells that differentiate into types I-III cells (Miura et al., 2014). The specific type of taste cells responsible for salt taste reception remains a matter of debate.

## Amiloride-sensitive and -insensitive salty taste

**Figure 1:**
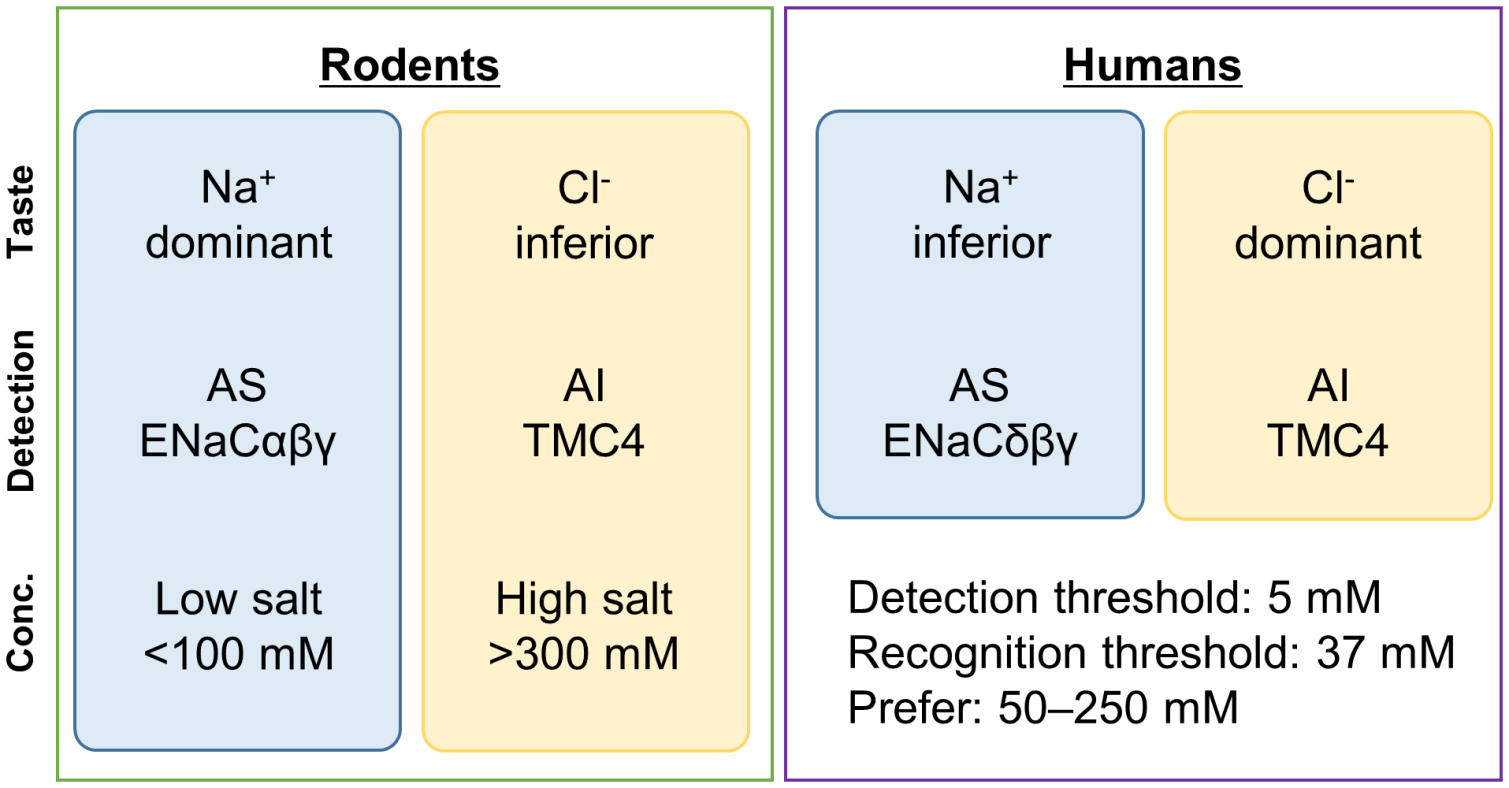
Summary of sensing of a typical salty taste substance, sodium chloride (NaCl). Some differences exist in the reception of salty taste between rodents and humans. In humans, the taste of Cl^−^ is more dominant than that of Na^+^. In rodents, ENaC*αβγ* is involved in AS, while in humans, ENaC*δβγ* is involved. In contrast, TMC4 is involved in AI in both cases. In rodents, ENaC*αβγ* is mainly involved in the reception of low concentrations of salty taste (<100 mM) (Oka et al., 2013), while TMC4 is believed to be involved in the reception of high concentrations (>300 mM) (Kasahara et al., 2021a). Generally, in humans, the detection and recognition thresholds for NaCl are 5 mM and 37 mM, respectively (Webb et al., 2015), and NaCl concentrations of 50–250 mM are preferred (Chamoun et al., 2019). The concentration ranges in which ENaC*δβγ* and TMC4 are sensorially involved need to be examined in the future. Conc., concentration; AS, amiloride-sensitive; AI, amiloride-insensitive; TMC4, Transmembrane channel-like 4; ENaC, epithelial sodium channel.

Salty taste is divided into amiloride-sensitive and -insensitive salty tastes based on the difference in sensitivity to the diuretic amiloride (Brand et al., 1985; Kretz et al., 1999) (Figure 1).

Because amiloride is a specific inhibitor of the epithelial sodium channel (ENaC), the receptor for the amiloride-sensitive salty taste response is thought to be ENaC. ENaC is a non-voltage-gated sodium channel (Stockand et al., 2008). Each ENaC subunit has two transmembrane domains, and ENaC functions as a heterotrimer composed of either ENaC*αβγ* or ENaC*δβγ* (Stockand et al., 2008; Noreng et al., 2018). ENaC*αβγ* predominates in mouse taste cells, whereas ENaC*δβγ* is more common in human taste cells (Kretz et al., 1999; Stähler et al., 2008) (Figure 1). In ENaC*α*-deficient mice, both the chorda tympani nerve response and licking response for low sodium concentrations are significantly reduced (Chandrashekar et al., 2010). It has also been reported that these responses are transmitted to the central nervous system through calcium homeostasis modulator (CALHM)1/CALHM3 channels expressed in type II cells (Nomura et al., 2020). However, it has been suggested that in fungiform papillae deeply involved in amiloride-sensitive salty taste reception, each subunit is expressed exclusively and may not function as the heterotrimeric ENaC*αβγ* (Lossow et al., 2020). It is widely acknowledged that amiloride significantly suppresses salty taste reception in mice through the ENaC*αβγ*, nevertheless determining the exact stoichiometry of ENaC subunits in taste cells remains a challenge for future research.

Regarding the effect of amiloride on salt taste reception in humans, it has been reported that human salt taste reception is not significantly influenced by amiloride in sensory tests (Desor and Finn, 1989; Halpern and Darlington, 1998). Additionally, cell experiments have shown that the 50% inhibitory concentration (IC_50_ ≓ 2.7 μM) of ENaC*δβγ*, which is predominant in humans, is approximately 25 times higher than that of ENaC*αβγ* (IC_50_ ≓ 0.11 μM) (Waldmann et al., 1995; Ji et al., 2012). These suggest that ENaC*δβγ* is less sensitive to amiloride and amiloride-sensitive salty taste plays a minor role in salt taste reception in humans. Therefore, in order to elucidate the mechanism of salt taste reception in humans, it is important to understand the pathways that are insensitive to amiloride.

It has been suggested that type II and III cells are involved in the amiloride-insensitive salt response (Oka et al., 2013). Double mutant mice, generated by crossing *transient receptor potential cation channel subfamily M member 5* (*TRPM5*) knock out (KO) mice and *polycystic kidney disease 2-like 1* (*PKD2L1*)-TeNT mice, showed significant reduction in amiloride-insensitive sodium response compared to wild type (WT) and single mutant mice (Oka et al., 2013). *TRPM5* is involved in the transmission of taste signals in type II cells, and *PKD2L1* is a molecular marker of type III cells (Zhang et al., 2003; Ishimaru et al., 2006; LopezJimenez et al., 2006). The *PKD2L1*-TeNT indicated that the neural connections of *PKD2L1*-expressing taste cells were blocked by diphtheria toxin (Huang et al., 2006). However, the specific receptors involved in these responses remains unknown.

Besides, it has been reported that the taste variant of *transient receptor potential cation channel subfamily V member 1* (*TRPV1*t), a splicing variant of *TRPV1* is involved in amiloride-insensitive sodium responses (Lyall et al., 2004). TRPV1 is a non-selective cation channel and mediated pain, hot temperature, and pungent taste (Tominaga et al., 1998; Tominaga, 2005). However, the results of tissue staining and gene expression analysis have shown that *TRPV1* is expressed in sensory nerve terminals distributed around the taste buds, but not in taste cells (Ishida et al., 2002; Kido et al., 2003; Sukumaran et al., 2017). Additionally, some reports on salty taste reception using *TRPV1* KO mice do not support the involvement of *TRPV1* in salty taste reception (Treesukosol et al., 2007; Smith et al., 2012). Hence, the specific receptors responsible for amiloride-insensitive salty taste remains unidentified.

## Taste of chloride ion

The majority of studies on salt taste reception have focused on cations, particularly sodium ions. The inhibitory effect of amiloride on salt taste reception in rodents is highly potent, and the target molecule, ENaC, is well known. Therefore, research on amiloride-sensitive salty taste remains a mainstream approach in the study of salt taste reception. On the other hand, research on chloride taste regarding salt taste reception is limited. However, studies using human sensory evaluation have shown that Na-acetate and Na-ascorbate Na-citrate have a weaker salt taste compared to NaCl at the same concentration, implying that chloride ions are involved in salt taste reception (Van der Klaauw and Smith, 1995). Previously, Ye et al. referred to the phenomenon where sodium salts other than NaCl have a weaker salty taste as the “anion paradox” or “anion effect” (Ye et al., 1991). They hypothesized that this occurs because large anion block the depolarization of taste cells triggered by influx of sodium ion (Ye et al., 1991). Until recently, another widely accepted hypothesis was that chloride ion channels were not involved in salt taste reception in taste cells, and chloride ions were thought to flow through tight junctions between taste cells (Elliott and Simon, 1990; Ye et al., 1993).

However, it has been reported recently that taste cells that respond strongly to salty stimulus are classified into two types: anion-insensitive (respond equally to the same concentration of NaCl) and anion-sensitive (respond differently to NaCl and Na-gluconate) (Lewandowski et al., 2016). This suggests that salt taste receptor cells respond differently to various anions. In addition, another study showed that amiloride-insensitive salt taste responses depend on chloride ions rather than sodium ions, and that the receptor may exist in taste cells (Roebber et al., 2019). These reports have updated old hypotheses regarding the anion effect, strongly suggesting that this effect may be mediated by chloride channels involved in salt taste reception in taste cells.

## Expression of transmembrane channel-like 4 (TMC4) in the taste bud

Based on this background, we focused on the amiloride-insensitive pathway and attempted to identify new molecules involved in human salt taste reception. First, we screened candidate molecules. The amiloride-insensitive salt pathway is prominently observed in the glossopharyngeal nerve, which connects the taste bud tissue of the circumvallate papillae and foliate papillae at the posterior of the tongue (Formaker and Hill, 1991; Chandrashekar et al., 2010; Oka et al., 2013). Therefore, we conducted a comparative analysis of gene expression in the epithelium of circumvallate papillae with taste buds and the epithelium at the edges of circumvallate papillae without taste buds (Kasahara et al., 2021a), and obtained 1,120 candidate molecules, including transmembrane channel-like (TMC) family. TMC proteins are membrane proteins that are conserved from *C. elegans* to humans. In mammals, eight TMC family members have been identified (Hahn et al., 2009). In mice, TMC1 and TMC2 are known that are mechanosensitive cation channels involved in hearing (Kawashima et al., 2011). In addition, it has been reported that TMC1 functions as a sodium-sensitive membrane protein in *C. elegans* (Chatzigeorgiou et al., 2013). Of the eight TMC members, only three molecules *Tmc4*, *Tmc5*, and *Tmc7* were included in the candidate molecules. The expression level of *Tmc4* was the strongest among them.

Subsequently, the localization of *Tmc* mRNAs expression in taste buds was observed by *in situ* hybridization (Kasahara et al., 2021a). The *Tmc4* signals were specific to the taste bud tissues of circumvallate and foliate papillae, and strong signals were observed. On the other hand, the signal of *Tmc4* in taste buds of fungiform papillae was very weak.

## Molecular properties of TMC4

The function of TMC4 was analyzed by expressing mouse TMC4 (mTMC4) in cultured cells using a whole-cell patch clamp method (Kasahara et al., 2021a). When step pulses were applied to a cell expressing mTMC4, large outward currents were observed at positive potentials, which increased in a voltage-dependent manner. Furthermore, the effects of intracellular and extracellular ion compositions and the administration of various inhibitors on the current, were examined. The mTMC4-mediated current was not affected by intracellular and extracellular cations or amiloride, but was significantly reduced by the addition of several anion channel inhibitors. In addition, the mTMC4-mediated outward current did not completely disappear when NaCl was replaced with Na-gluconate in the bath solution, suggesting that mTMC4 functions as a voltage-dependent chloride channel with large pore.

## Salt taste reception in *Tmc4*-deficient mice

Next, we observed salt responses in *Tmc4*-deficient mice using a taste nerve recording and a licking behavior test (Kasahara et al., 2021a; Narukawa et al., 2024). In the glossopharyngeal nerve, *Tmc4*-deficient mice exhibited a significant reduction in responses to high concentrations of NaCl and KCl compared with wild type (WT) mice. In contrast, in the chorda tympani nerve, no significant difference was observed in responses to NaCl and KCl at any concentration (Kasahara et al., 2021a). In the licking test, *Tmc4-*deficient mice exhibited a significant decrease in sensitivities to multiple chloride salts such as NaCl, KCl, and NH_4_Cl (Narukawa et al., 2024). Furthermore, there was no significant difference in the responses to sweet, umami, bitter, and sour taste stimulus between WT and *Tmc4-*deficient mice. These suggests that TMC4 plays as a key molecule in salt taste reception.

## Insight into the role of chloride ion in salt taste reception

Generally, chloride ion influx regulates membrane potential, promoting repolarization (Huang et al., 2012). The molecular simulation using a model taste cell showed that TMC4 presence shortens action potential duration in response to salt, increasing firing frequency under continuous stimulation (Kasahara et al., 2021a). We hypothesize that TMC4 accelerates the salt taste signaling cycle by allowing chloride ions to enter taste cells, generating new salt taste signals.

## Evolutionary viewpoint of TMC genes

Among the TMC family, TMC1 and TMC2 proteins are conserved across many vertebrates, including the green sea turtle, budgerigar, zebrafish, and pufferfish (Keresztes et al., 2003; Jia et al., 2020). In contrast, there are few reports on TMC4 expression in non-mammals. Although pufferfish is the only non-mammalian organism where TMC4 expression has been confirmed, the presence of TMC4, which is involved in salt taste reception, in both terrestrial organisms such as humans and mice, where salt is a limited resource and marine organisms like pufferfish, where salt is abundant, may provide insights into the mechanisms of terrestrial adaptation from an evolutionary medicine perspective (Keresztes et al., 2003).

## Application of TMC4 for reducing salt intake

Our findings strongly suggest that TMC4 plays a role in amiloride-insensitive salty taste and that chloride ion is the primary component of amiloride-insensitive salty taste (Figure 1).

Some sensory tests have reported that human salt taste reception is influenced by temperature, pH, and non-steroidal anti-inflammatory drugs (Pangborn et al., 1970; McBurney et al., 1973; Keast and Breslin, 2003; Schiffman et al., 2000). We confirmed that human TMC4 (hTMC4) responds to these stimuli in a manner similar to human sensory evaluation (Kasahara et al., 2021b,c). Furthermore, utilizing the structure of L-arginine hydrochloride, known as a salt taste enhancer, we discovered a novel salt taste enhancer, 3-guanidinylpropanol hydrochloride (3GPrOH), and demonstrated that 3GPrOH significantly enhances hTMC4-mediated currents, thereby enhancing salty taste (Lee, 1992; Sakurai et al., 2015; Kasahara et al., 2022). In addition, some researchers report that salt taste enhancing peptides enhance the salty taste via TMC4, based on the structure of TMC4 created *in silico* method using artificial intelligence programs (Shen et al., 2022; Bu et al., 2024; Zhang et al., 2024; Wang et al., 2024). These results suggest that TMC4 is involved in salt taste reception in humans and can be applicable to the development of salt taste enhancers.

As indicated in the introduction, in recent years, it has been reported that excessive sodium intake is linked to the various health risk. In 2012, the WHO strongly recommended a daily NaCl intake target of 5 g for adults (World Health Organization, 2012). According to this, many countries have been exploring various approaches to reduce sodium intake. As a result, efforts to reduce salt intake have included the establishment of new nutritional guidelines, consumer education on salt, labeling the sodium content on food packaging, requests for salt reduction from food companies, and the introduction of salt taxes (Santos et al., 2021; World Health Organization, 2023). However, even with these mandatory policies, there is still a considerable gap in achieving WHO’s goals (Alonso et al., 2021; Looi, 2023; World Health Organization, 2023).

Some epidemiological studies suggest a strong positive correlation between salt intake and blood pressure (Robinson et al., 2019). Hypertension is a contributing factor to heart disease and chronic kidney disease (CKD). It has been reported that CKD patients suffer from oral diseases and have dulled taste sensitivity, leading them to prefer high concentrations of salt (Okuno-Ozeki et al., 2024). In addition to the taste papillae, TMC4 is highly expressed in the duodenum, colon, and small intestine, but is barely expressed in the brain (Fagerberg et al., 2014). Analysis of TMC4 expression and mutations in the oral cavity of subjects with an addictive preference for salt and CKD patients may provide valuable insights for developing treatments for these conditions.

Exploring substances that effectively reduce salt intake using TMC4, and elucidating the salt taste reception mechanism with TMC4, will aid in achieving this challenging goal. TMC4 is the first molecule reported to be involved in chloride taste reception. Research involving TMC4 is expected to contribute to improving our quality of life.
